# Obituary

**Published:** 1918-01

**Authors:** 


### OBITUARY

Edward C. Harmeyer, formerly a member of S. A. Crocker & Co., but later of Harmeyer and Brand, died on November 8th at his home in Cincinnati, after a short illness.

